# Spontaneous Uterine Cornual Rupture in the Second Trimester of Dichorionic-Diamniotic Twin Pregnancy

**DOI:** 10.1055/a-2946-0857

**Published:** 2026-09-08

**Authors:** Britney Margheim, Timothy Nguyen, Mathias Wiebe, Melton Zheng, Saurabh Kumar, Roufan Yoa

**Affiliations:** 1 472547 Kansas City University College of Osteopathic MedicineKansas CityMissouriUnited States; 2 155229 Touro University California College of Osteopathic MedicineVallejoCaliforniaUnited States; 3 657704 American Canadian School of Medicine at DominicaPicardSaint John ParishDominica; 4 25112 Desert Regional Medical CenterPalm SpringsCaliforniaUnited States; 5 23335 Loma Linda University Medical CenterLoma LindaCaliforniaUnited States

**Keywords:** cornual uterine rupture, twin pregnancy, spontaneous uterine rupture, second-trimester pregnancy, hemoperitoneum, acute abdomen in pregnancy, exploratory laparotomy

## Abstract

**Background** Uterine rupture is a rare but life-threatening obstetric emergency, usually associated with labor in patients with a prior cesarean delivery. Spontaneous rupture remote from a uterine scar, particularly at the cornua in the second trimester, is exceedingly uncommon.

**Case** We report a 30-year-old gravida 2 para 1 at 25 ^3/7^ weeks’ gestation with a dichorionic–diamniotic twin pregnancy who presented with abrupt abdominal pain and hemoperitoneum of unclear etiology. Despite reassuring imaging, progressive maternal deterioration and non-reassuring fetal status prompted emergent laparotomy and cesarean delivery. A 3 cm × 3 cm full-thickness left cornual rupture was identified.

**Conclusion** In pregnant patients with unexplained hemoperitoneum, clinical status must override reassuring imaging, and prompt surgical intervention is essential.

## Introduction

Uterine rupture is a rare but life-threatening obstetric emergency associated with significant maternal morbidity and perinatal mortality. 1 Most cases occur in the setting of a prior uterine scar, particularly during labor. 2 The reported incidence is approximately 0.38 per 10,000 deliveries in patients without a prior cesarean delivery, compared with 21.1 per 10,000 in those with a history of cesarean delivery. 2

Although most ruptures occur along prior uterine incisions, rupture at the uterine cornua represents a particularly rare and poorly characterized entity. Epidemiologic data regarding cornual rupture, particularly outside of ectopic pregnancy, remain limited. 3

Here, we report a rare case of a second-trimester twin gestation complicated by spontaneous cornual uterine rupture in the setting of a normally implanted intrauterine pregnancy without identifiable risk factors and no evidence of active bleeding on computed tomography (CT), highlighting the diagnostic challenges and clinical considerations associated with this presentation.

## Case Presentation

A 30-year-old gravida 2 para 1 female with a known history of a prior low transverse cesarean delivery presented at 25 ^3/7^ weeks’ gestation with a dichorionic–diamniotic twin pregnancy complicated by acute onset of severe abdominal pain. Initial evaluation at an outside hospital revealed hemoperitoneum of unclear etiology on imaging. Pelvic examination demonstrated a closed cervix without evidence of vaginal bleeding. She was subsequently transferred for higher-level care.

**Fig. 1:**
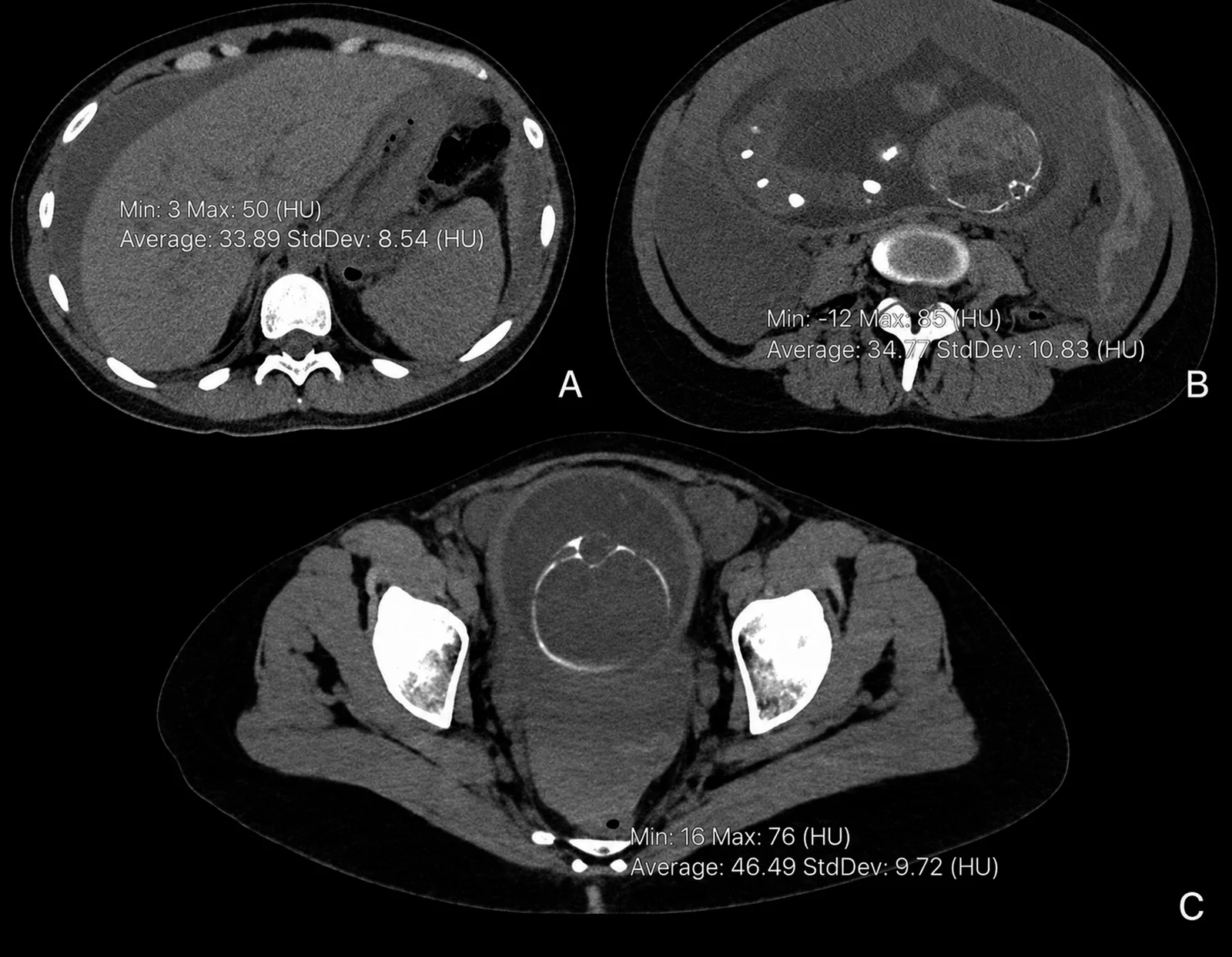
Axial non-contrast CT images of the abdomen and pelvis demonstrating hemoperitoneum at three levels: perihepatic region measuring 25.89 ± 8.54 HU ( **A** ), mid-abdomen at the level of the kidneys measuring 24.77 ± 10.93 HU ( **B** ), and the pelvis at the level of the bladder measuring 46.49 ± 9.72 HU ( **C** ). The higher attenuation of dependent pelvic fluid is consistent with acute clot formation, while perihepatic and mid-abdominal values reflect subacute or diluted blood products, in keeping with hemoperitoneum.

Upon arrival, imaging with non-contrast CT of the abdomen and pelvis demonstrated a moderate volume hemoperitoneum with preservation of uterine contour and no radiographic evidence of uterine disruption ( Fig. 1 ). A focused assessment with sonography for trauma (FAST) examination was positive for free fluid in all four abdominal quadrants. Contrast imaging was initially deferred. The patient was hemodynamically stable, and fetal assessment was overall reassuring. She was managed expectantly with close monitoring.

**Fig. 2:**
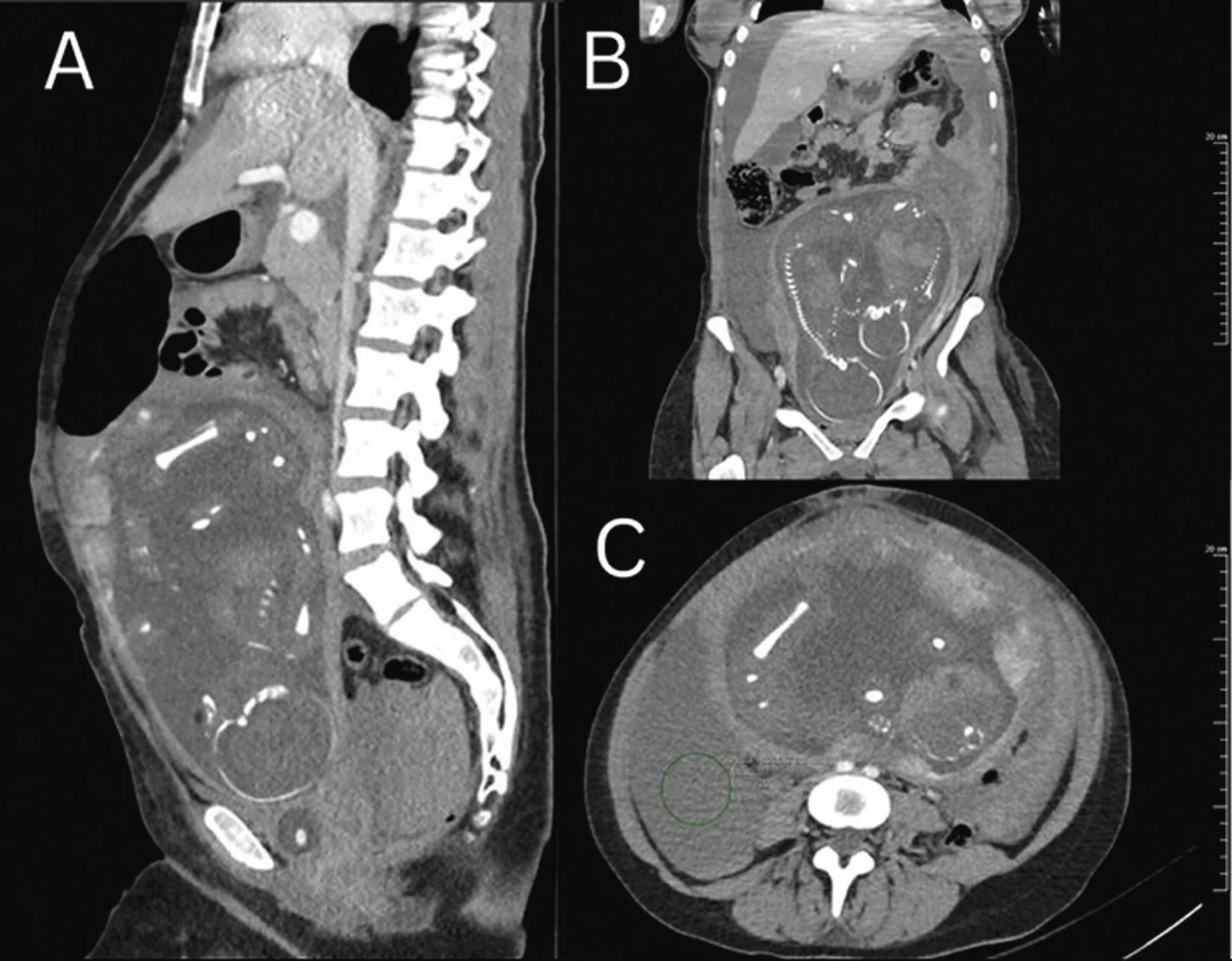
Sagittal ( **A** ), coronal ( **B** ), and axial ( **C** ) contrast-enhanced CT images of the abdomen and pelvis in the patient with twin gestation. The sagittal image demonstrates a hyperdense fluid collection in the presacral space ( **A** ). The coronal image demonstrates a twin gestation with periuterine hyperdense free fluid consistent with intraperitoneal hemoperitoneum; no intrauterine hemorrhage is identified ( **B** ). The axial image re-demonstrates intraperitoneal hemoperitoneum with layering in the paracolic gutters ( **C** ), consistent with hemoperitoneum in the setting of an ongoing twin pregnancy.

Overnight, her condition worsened with severe diffuse abdominal pain, distension, and clinical concern for ongoing hemorrhage. The patient subsequently consented to CT angiography with contrast, which demonstrated persistent moderate hemoperitoneum without evidence of active contrast extravasation ( Fig. 2 ). Fetal monitoring deteriorated, with persistent Category II tracings and recurrent deep decelerations in Twin A. Given worsening maternal and fetal status, emergent exploratory laparotomy and classical cesarean delivery were performed.

Upon entry into the peritoneal cavity, significant hemoperitoneum was evacuated. Systematic inspection of the uterus revealed an intact anterior wall and lower uterine segment, including a well-healed prior low transverse hysterotomy site. After the delivery of both fetuses, intra-abdominal exploration revealed a 3 cm × 3 cm full-thickness rupture at the left uterine cornua, with slow bleeding identified as the source of hemoperitoneum. The defect was repaired, achieving hemostasis.

The patient stabilized postoperatively and was discharged on postoperative day 5. One neonate survived; the second did not survive despite resuscitation.

## Discussion

Uterine rupture is most commonly associated with labor in patients with a prior cesarean delivery. 1 In contrast, spontaneous rupture remote from a prior uterine scar, particularly at the cornua and during the second trimester, is exceedingly rare. 4

A review of published cases of spontaneous uterine rupture ( Table 1 ) revealed that nearly all reported cases are associated with at least one identifiable risk factor, most commonly prior uterine instrumentation, such as cesarean delivery, dilation and curettage, or myomectomy, or conditions predisposing to myometrial compromise, including ectopic cornual pregnancy, adenomyosis, abnormal placentation, in vitro fertilization (IVF), or twin–twin transfusion syndrome.

**Table 1:** Summary of the clinical characteristics and rupture sites across the 11 cases identified in the literature review (search performed on April 6, 2026).

Author	Year	Gestational age at presentation (wk)	Gestation type	Risk factors	Site of uterine rupture
Paradise et al 6	2015	26	Dichorionic/diamniotic	Open appendectomy, laparoscopic bilateral salpingectomy, heterotopic interstitial pregnancy, in vitro fertilization	Left cornua
Yang et al 4	2021	27	Singleton	Oligohydramnios	Right cornua
Mizutamari et al 7	2014	32	Singleton	N/A	Left cornua
Liao et al 5	2009	13 ^3/7^	Not reported	Previous cornual pregnancy	Left upper cornea
Doger et al 14	2013	32	Dichorionic/diamniotic	In vitro fertilization (IVF)	Right posterior utero-ovarian vein
Hamar et al 15	2003	20	Singleton	Previous cesarean delivery	Lower uterus posterior to bladder
Hawkins et al 16	2018	21 ^2/7^	Singleton	Multiple myomectomies	Left aspect of uterine fundus
Smid et al 17	2015	21 ^3/7^	Monochorionic/diamniotic	Stage-1 twin–twin transfusion syndrome (TTTS), previous cesarean delivery, dilation and curettage (D&C), intrauterine device placement, multiple 1st trimester miscarriages	Posterior serosa of lower left uterine segment
Bhikha-Kori et al 18	2017	21	Monochorionic/diamniotic	TTTS, previous cesarean delivery, Hemolysis, Elevated Liver Enzymes, Low Platelets (HELLP) syndrome, intrauterine growth restriction	Right uterus at previous scar site
Saciragic et al 19	2015	32	Dichorionic/diamniotic	Previous D&C	Right lateral uterine wall medial to right uterine artery
Smith et al 10	2021	31	Dichorionic/diamniotic	Previous cesarean delivery	Uterine fundus
Li et al 8	2021	29 ^6/7^	Dichorionic/diamniotic	Suspected endometriosis, adenomyosis	Posterior wall of uterus near right sacral ligament at site of adenomyoma
Mannini et al 11	2016	15	Singleton	Previous miscarriage, D&C, appendectomy	Uterine fundus

Spontaneous cornual rupture is exceedingly rare, with only four cases reported: following a prior cornual pregnancy 5 ; in a patient with multiple risk factors, including prior laparoscopic bilateral salpingectomy, heterotopic interstitial pregnancy, and IVF 6 ; in association with oligohydramnios 4 ; and in a patient without identifiable risk factors. 7 In that case, however, imaging demonstrated protrusion of the amniotic cavity through a uterine wall defect, a feature not observed in our patient. 7

**Fig. 3:**
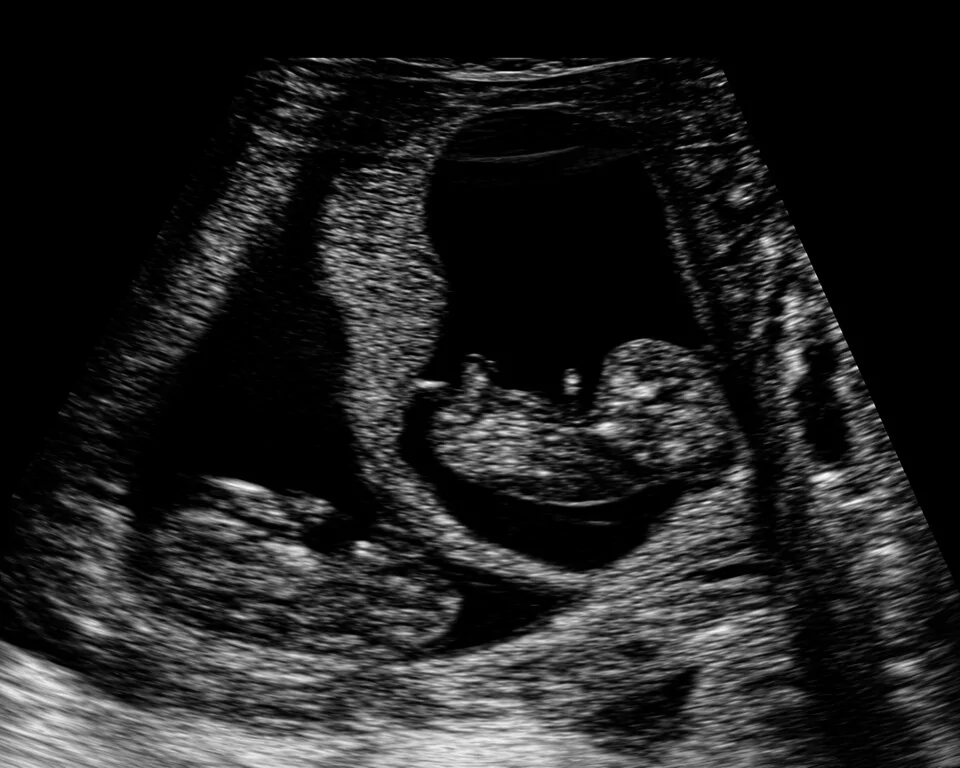
First-trimester ultrasound demonstrating a twin intrauterine pregnancy with a thick dividing membrane and probable twin-peak sign. No angular or cornual implantation identified.

Cornual rupture is more commonly described in the setting of cornual ectopic pregnancy, in which first-trimester ultrasound typically demonstrates the interstitial line sign and an eccentrically located gestational sac surrounded by a thin myometrial mantle. 3 These findings were absent on our patient’s initial imaging, making cornual ectopic pregnancy unlikely at presentation ( Fig. 3 ). This case therefore represents spontaneous cornual rupture in a normally implanted intrauterine pregnancy without identifiable risk factors, further highlighting the rarity of this presentation.

The etiology of rupture in this patient remains unclear. Although the cornual region is structurally and vascularly predisposed to significant hemorrhage when disrupted, there was no evidence of prior ectopic implantation, residual tissue, cornual distension, or congenital uterine anomaly. Spontaneous rupture at a non-scar site in the absence of labor has been hypothesized to result from focal myometrial weakness, Müllerian duct anomalies, or localized structural defects. 4 Importantly, the rupture site did not correspond to the patient’s prior cesarean scar, supporting a non-iatrogenic mechanism. Additionally, there was no extrusion of fetal or placental tissue through the defect, and the bleeding appeared slow and venous in nature, contributing to the atypical presentation. Notably, the patient lacked nearly all recognized risk factors for rupture at a non-scar site. 4 8

Multiple gestation pregnancy has been shown to increase the risk of uterine rupture, even in unscarred uteri, and large population-based data from the United States identify it as one of the strongest risk factors in patients without a prior cesarean delivery. 9 In the literature reviewed, multigestational pregnancies are frequently represented, accounting for 54% of cases. Twin gestation results in significant uterine enlargement and increased intrauterine pressure, leading to physiologic overdistension that may predispose the uterus to rupture, particularly at structurally weaker areas. 8 10 This overdistention due to twin pregnancy may have been the only possible identifiable risk factor for uterine rupture at the cornua.

Diagnosis of uterine rupture remains challenging, particularly in earlier gestation and in atypical presentations, as clinical manifestations are often nonspecific and may evolve over time. 11 Presentation also varies by location of the defect. Rupture involving the lower uterine segment is more likely to present with vaginal bleeding, whereas fundal or cornual rupture typically results in intraperitoneal hemorrhage, which can obscure the diagnosis. 12 As a result, definitive diagnosis is frequently established only at the time of surgical exploration. 12 Although imaging can provide supportive information, its sensitivity is limited. Ultrasonography may demonstrate uterine wall disruption, but detection generally requires a sizable defect, and posterior or cornual lesions are particularly difficult to visualize. 11 This limitation is illustrated in the present case, where the absence of vaginal bleeding, lack of labor, and preserved uterine contour on imaging reduced initial suspicion for rupture. Despite moderate hemoperitoneum on ultrasound and CT, no definitive source of bleeding, radiographic evidence of uterine rupture, or active contrast extravasation was identified. Intraoperatively, however, a 3 × 3 cm full-thickness rupture at the left uterine cornua with slow, ongoing bleeding was found to be the source. It is possible the site was not actively bleeding at the time of imaging, underscoring the limited sensitivity of radiographic modalities in detecting cornual rupture.

Hemoperitoneum during pregnancy is an uncommon but serious condition that demands rapid recognition, stabilization of the patient’s hemodynamic status, and frequent surgical management. 13 In most cases (~93%), treatment involves exploratory laparotomy, often performed alongside cesarean delivery when the fetus has reached viability. 13 In this case, unexplained hemoperitoneum without a clear source on imaging contributed to diagnostic uncertainty and highlights that worsening clinical status should prompt early surgical intervention despite inconclusive imaging. The decision to proceed with emergent classical cesarean delivery and exploratory laparotomy was guided by both maternal and fetal status, with the fetuses suspected to be viable at 25 weeks’ gestation.

## Conclusion

Spontaneous cornual uterine rupture in the second trimester of a normally implanted intrauterine pregnancy without identifiable risk factors is rare and may present without classic features or clear radiographic findings. This case highlights that imaging can be falsely reassuring and should not outweigh worsening clinical status. In the setting of maternal instability or evolving fetal compromise, early multidisciplinary involvement and a low threshold for exploratory laparotomy are essential. Ultimately, this case underscores that rare entities such as spontaneous cornual rupture should remain within the differential diagnosis for second-trimester abdominal emergencies, particularly in multiple gestations, and that timely surgical intervention may be both diagnostic and life-saving.
